# Long-term stability of hairline landmarks in treated frontal fibrosing alopecia: A retrospective cohort study

**DOI:** 10.1016/j.jdin.2026.02.011

**Published:** 2026-03-20

**Authors:** Raymond Z. Ezzat, Aubrey Martin, Meshi Paz, Mack Su, Caroline Kreytak, Ogechi Ezemma Siaw, Maryanne M. Senna

**Affiliations:** a Department of Dermatology, Lahey Hospital & Medical Center, Burlington, Massachusetts; b Department of Dermatology, Harvard Medical School, Boston, Massachusetts; c Department of Dermatology, University of Pennsylvania, Philadelphia, Pennsylvania

**Keywords:** cicatricial alopecia, FFA, frontal fibrosing alopecia, hairline measurements, scarring alopecia, treatment

*To the Editor:* Frontal fibrosing alopecia (FFA) is a lymphocytic cicatricial alopecia characterized by progressive recession of the frontotemporal area.^1^^,^^2^ Longitudinal, site-specific hairline measurements are rarely reported, so the degree of stabilization achievable with routine therapy is unclear. Thus, we performed a retrospective cohort study to quantify long-term changes in standardized hairline landmark distances and perifollicular activity scores in women with FFA managed in a tertiary hair clinic.

Consecutive patients (2015-2020) at the same tertiary hair clinic with dermatologist-confirmed FFA who had standardized hairline measurements at baseline and at least 3 years of follow-up were included. FFA diagnosis was made by the same board-certified dermatologist (MMS) based on clinical features, trichoscopy, and, if needed, confirmatory biopsy. All hairline measurements were performed by the same experienced hair dermatologist (MMS) using a flexible tape and a standardized protocol, with the patient in neutral gaze.

Distances in centimeters were measured from 5 anatomic landmarks outlined in Table I. Negative change indicates a decrease in distance; positive change indicates a worsening of hairline recession.^3^

**Table I:** Standardized frontal hairline landmark definitions and measurement protocol used for tape measurements in frontal fibrosing alopecia

Landmark	Reference facial landmark(s)	Measurement description (cm)∗	Special considerations
Glabella midline	Lower glabellar crease; frontal hairline at the midline	Vertical distance from the lower glabellar crease to the frontal hairline at the midline measured with a flexible tape	None
Right midbrow	Superior margin of right midbrow at midpupillary line; frontal hairline	Vertical distance from the superior margin of the right midbrow at the midpupillary line to the frontal hairline	If eyebrow hair is thinned or absent, use the bony superior orbital rim at the right midpupillary point as the reference
Left midbrow	Superior margin of left midbrow at midpupillary line; frontal hairline	Vertical distance from the superior margin of the left midbrow at the midpupillary line to the frontal hairline	If eyebrow hair is thinned or absent, use the bony superior orbital rim at the left midpupillary point as the reference
Right lateral canthus	Right lateral canthus; frontal hairline	Vertical distance from the right lateral canthus to the frontal hairline	None
Left lateral canthus	Left lateral canthus; frontal hairline	Vertical distance from the left lateral canthus to the frontal hairline	None

∗ Negative change in distance indicates a shorter landmark-to-hairline distance (movement of the frontal hairline toward the facial landmark, interpreted as improvement). Positive change indicates a longer distance (worsening hairline recession).

Perifollicular erythema and scale were graded on a scale of 0 (none) to 3 (severe) at each visit using trichoscopy by the same hair dermatologist (MMS). The analyses were predefined: paired *t* tests were used to compare hairline distances, and Wilcoxon’s signed rank tests were used for ordinal signs, with a 2-sided α level of 0.05. Treatment was at the discretion of clinicians and was heterogeneous by design.

Thirty-seven women (mean age, 66.9 years) met the criteria. The mean duration between measurements was 4.78 years (range, 3.0-8.33 years), and the average disease duration was 8.01 years (range, 3.6-15.5 years). Treatment regimens commonly included topical immunomodulators, hydroxychloroquine, and adjunctive low-dose oral or topical minoxidil.

Hairline distances improved at 4 of the 5 measured sites, with statistical significance observed only at the midbrow landmarks: right midbrow decreased by 0.27 cm (*P* = .0208) and left midbrow by 0.38 cm (*P* = .0004). Nonsignificant reductions were observed at the lateral canthi: right by 0.36 cm (*P* = .3922) and left by 0.13 cm (*P* = .7011) (Table II). The midline of the glabellar region showed a slight increase (+0.15 cm; *P* = .2601), which was not statistically significant. There was an improvement in clinical activity, evidenced by a reduction in perifollicular erythema (−1.19 points; *P* = 2.25 × 10^−6^) and scaling (−0.51 points; *P* = .015).

**Table II:** Cohort characteristics and paired changes in standardized hairline landmarks and perifollicular activity in treated frontal fibrosing alopecia

Characteristic	Value
No. of patients with both time points	37
Mean age (y)	66.9
Mean duration of disease (y)	8.01
Mean time between measurements (y)	4.78
Hairline measurement changes	
Right lateral canthus (cm)	−0.36 (*P* = .3922)
Right midbrow (cm)	−0.27 (*P* = .0208)∗
Lower glabellar crease (cm)	+0.15 (*P* = .2601)
Left midbrow (cm)	−0.38 (*P* = .0004)∗
Left lateral canthus (cm)	−0.13 (*P* = .7011)
Perifollicular inflammation markers	
Erythema	−1.19 (*P* = 2.25 × 10^−6^)∗
Scale	−0.51 (*P* = .015)∗

Negative Δ indicates movement toward the brow (decrease in distance). Values are mean change (follow-up − baseline) unless noted; *P* values from paired *t* tests for distances and Wilcoxon’s signed rank for ordinal signs.

∗ Statistical significance (*P* < .05).

Over approximately 5 years, the treatment of FFA in routine practice was associated with minor, site-specific improvements in the midbrow area and a reduction in perifollicular activity. The anatomic heterogeneity and greater sensitivity of the midbrow suggest that these landmarks may serve as more reliable indicators of positional changes than the lateral canthi. Although absolute distance changes were modest (<0.5 cm), in a scarring alopecia that otherwise tends to progress, they support stabilization with minor improvement as a realistic treatment goal.^4^

Limitations of this study include its retrospective design, heterogeneous treatment regimens, absence of an untreated control group, and lack of formal interrater reliability assessments for tape measurements.

## Conflicts of interest

None disclosed.
