# Correction to ‘Long-term stability of hairline landmarks in treated frontal fibrosing alopecia: A retrospective cohort study’ [*JAAD International* 2026 June;(26)34-36]

**DOI:** 10.1016/j.jdin.2026.05.001

**Published:** 2026-05-12

Raymond Z. Ezzat,^a^ Aubrey Martin,^a^ Meshi Paz,^a^ Mack Su,^a^ Caroline Kreytak,^a^ Ogechi Ezemma Siaw,^c^ Maryanne M. Senna^a,b^

^a^Department of Dermatology, Lahey Hospital & Medical Center, Burlington, Massachusetts

^b^Department of Dermatology, Harvard Medical School, Boston, Massachusetts

^c^Department of Dermatology, University of Pennsylvania, Philadelphia, Pennsylvania

‘Long-term stability of hairline landmarks in treated frontal fibrosing alopecia: A retrospective cohort study’ was published with a typographical error in Table 1. The measurement description for ‘Right lateral canthus’ and ‘Left lateral canthus’ says ‘vertical distance’ when it should say ‘horizontal distance’.

A corrected version of the table is below.

**Table 1:** Standardized frontal hairline landmark definitions and measurement protocol used for tape measurements in frontal fibrosing alopecia

Landmark	Reference facial landmark(s)	Measurement description (cm)∗	Special considerations
Glabella midline	Lower glabellar crease; frontal hairline at the midline	Vertical distance from the lower glabellar crease to the frontal hairline at the midline measured with a flexible tape.	None.
Right mid-brow	Superior margin of right mid-brow at mid-pupillary line; frontal hairline	Vertical distance from the superior margin of the right mid-brow at the mid-pupillary line to the frontal hairline.	If eyebrow hair is thinned or absent, use the bony superior orbital rim at the right mid-pupillary point as the reference.
Left mid-brow	Superior margin of left mid-brow at mid-pupillary line; frontal hairline	Vertical distance from the superior margin of the left mid-brow at the mid-pupillary line to the frontal hairline.	If eyebrow hair is thinned or absent, use the bony superior orbital rim at the left mid-pupillary point as the reference.
Right lateral canthus	Right lateral canthus; frontal hairline	Horizontal distance from the right lateral canthus to the frontal hairline.	None.
Left lateral canthus	Left lateral canthus; frontal hairline	Horizontal distance from the left lateral canthus to the frontal hairline.	None.

∗ Negative change in distance indicates a shorter landmark-to-hairline distance (movement of the frontal hairline toward the facial landmark, interpreted as improvement). Positive change indicates a longer distance (worsening hairline recession).

Corresponding author: Maryanne M. Senna (maryanne.m.senna@lahey.org)

